# Impact of a national deworming campaign on the prevalence of soil-transmitted helminthiasis in Uganda (2004-2016): Implications for national control programs

**DOI:** 10.1371/journal.pntd.0006520

**Published:** 2018-07-05

**Authors:** Moses Adriko, Benjamin Tinkitina, Moses Arinaitwe, Narcis B. Kabatereine, Mariam Nanyunja, Edridah M. Tukahebwa

**Affiliations:** 1 Uganda Institute of Allied Health & Management Science (UIAHMS), School of Medical Entomology and Parasitology, Kampala, Uganda; 2 Vector Control Division, Ministry of Health, Kampala, Uganda; 3 Schistosomiasis Control Initiative, Department of Infectious Disease Epidemiology, Imperial College London, St Mary's Campus, Norfolk Place, London, United Kingdom; 4 World Health Organization, Kampala, Uganda; Ministère de la Santé Publique et de la Lutte contre les Endémies, NIGER

## Abstract

**Background:**

Soil-transmitted Helminths and Anemia potentially reduce and retard cognitive and physical growth in school-age children with great implications for national control programs in Africa. After 13 years of deworming and limited health education campaigns, a study was undertaken to evaluate the impact of deworming interventions on the prevalence and intensity of soil-transmitted helminthic infections in school-age children in Uganda.

**Methodology:**

A cross-sectional study was carried out in six regions of Uganda, where two districts were randomly selected per region based on the ecological zones in the country. Included in the study were the districts; Mpigi and Nakasongola from the Central; Nakapiripirit and Kotido from Karamoja; Arua and Yumbe from West Nile; Gulu and Alebtong from the North; Kaliro and Mbale from the East; Hoima and Bundibugyo in the West. Five schools were randomly selected from each district and in each school 50 children aged 6–14 years were randomly selected. Stool samples were taken each child and examined for the presence of helminthic infections. A short pretested questionnaire was administered to each participant to obtain their knowledge, attitude, and practice relating to STH infections, their control. General observations were made on environmental sanitation in the schools. The location of each school was geo-referenced using a GPS machine (Garmin^®^GPSMAP62, Garmin Ltd, Southampton, UK).

**Results:**

In total, 4,285 children were assessed including 719(16.82%) from central region, 718(16.80%) from eastern region, 719 (16.82%) from northern region, 689 (18.82%) from Karamoja region, 717(16.77%) from West Nile region and 723(16.91%) from western region. The average age of the children was 12.6 years with a standard deviation, SD 1.8 years and the minimum age was 6 years and upper age limit of 12 years. The percentage of boys (50.1%) and girls (49.9%) was comparable. 8.8% (95% CI; 8.0–9.7) were infected with at least any one STH species. Hookworm was the most prevalent (7.7%; 95% CI; 6.9–8.5) followed by whipworms (*Trichuris trichiura*) (1.3%; 95% CI; 1.0–1.7) and roundworms (*Ascaris lumbricoides*) (0.5%; 95% CI; 0.3–0.7). Some children had *Schistosoma mansoni*, 13.0% (95% CI; 12.0–14.0). All the children knew what soil transmitted helminths were (62.8%, 95% CI: 61.3–64.2) and most common knowledge of information were from; home (39%, 95% CI: 37.1–40.8), media (radio& newspaper)(11%, 95% CI: 9.8–12.2), school(65.7%, 95% CI: 63.9–67.5) and friends(11.5%, 95% CI: 10.3–12.7). Majority were aware of how one gets infected with soil transmitted helminths through; eating contaminated food (77.5%, 95% CI: 76.0–79.1), walking barefoot (59.6%, 95% CI: 57.8–61.5), drinking contaminated water (52.9%, 95% CI: 51.0–54.8), playing in dirty places (21.8%, 95% CI: 20.2–23.3) and dirty hands (2.3%, 95% CI: 1.7–2.9).

**Conclusion:**

Semi-annual deworming campaigns have proved effective in significantly reducing helminthic infections in most of the districts in Uganda. Regular evaluations are vital to assess impact of the interventions and guide programme implementation. Our data shows that the prevalence of infection has been reduced to a level where STH morbidity is no longer of public health importance in most districts surveyed.

## Author summary

Soil-transmitted Helminths potentially reduce physical growth and retard cognitive development in school-age children (SAC) with great implications for national control programs in Africa. In Uganda, baseline investigations between 1998 and 2002, indicated STH prevalence was over 60.0% in most districts, the commonest worms infections were *Hookworms*, *Ascaris* and *Trichuris*. Twice a year national deworming campaign was initiated in 2003 targeting aged 1–14 years. Over ten years of deworming campaigns, has reduced the overall STH prevalence to 8.8% in 2016. The findings suggest routine deworming campaigns reduce STH exposure and infections. Periodic program evaluations are key to determining the progress made in order to achieve the elimination targets by 2020.

## Introduction

Soil-transmitted Helminthic infections pose a great and often a silent burden of morbidity and mortality on poor populations in developing countries. The most common helminths infections are caused by roundworms (*Ascaris lumbricoides*), whipworms (*Trichuris trichiura*), and hookworms (*Necator americanus* and *Ancylostoma duodenal*). Worldwide estimates suggest that *A*. *lumbricoides* infects 1.221 billion people, *T*. *trichiura*, 795 million, and hookworms, 740 million [1].Between 1998 and 2003, the Vector Control Division of the MOH mapped schistosomiasis and soil-transmitted helminthiasis (STH) using the Kato Katz method[2]. The derived map, based on 23,627 individuals in 271 schools situated in 46 of the then 56 (82%) districts showed that STH prevalence was quite high, the prevalence of infection being over 60% in 35, of the 46 districts[3]. *A*.*lumbricoides* and *T*.*trichiura* were greatest in the south western districts with prevalence generally above 80%. Moderate prevalence occurred in selected central and eastern districts and there was a near absence of transmission in the northern districts[4]. By contrast, hookworm was homogeneously distributed throughout the country, exceeding 60% to 85% of the schools surveyed, but again the prevalence was quite low in the Karamoja region[4]. Thus according to WHO guidelines[5], virtually all districts in Uganda had a prevalence of any worm generally above 50% and the whole country fell into the category targeted for twice yearly treatment. A deworming campaign was initiated in 18 of the then 56 districts in 2003 by MoH supported by Schistosomiasis Control Initiative (SCI) but was scaled up to the national level in 2004[6]. Deworming activities are done once or twice a year mainly targeting PSAC and SAC but is also provided in antenatal clinics to expectant mothers. However, where LF is endemic the whole population receives albendazole as part of the LF treatment regimen with ivermectin and this given during one of the twice yearly deworming campaign.

### Prevalence and intensity of *STH* infections in school children

In terms of the disease burden in school-age populations in developing countries including Uganda [7], intestinal helminths infections rank first among the causes of all communicable and non-communicable diseases [8]. Field studies of Schistosomes and the major intestinal nematodes *Trichuris trichiura* and *Ascaris lumbricoides* repeatedly demonstrate that the intensity and prevalence of infection exhibit marked dependency on host age[9]. Peak levels of infection typically occur in hosts aged between 10 and 14 years in endemically infected communities [10]. Age-dependent patterns of infection prevalence are generally similar among the major helminth species, exhibiting a rise in childhood to a relatively stable asymptote in adulthood [11]. Epidemiological studies of STH infections have shown that the prevalence and intensity of infection are highest among children 4–15 years of age[3]. For the vast part of Uganda, a uniform treatment strategy involving school-based (for school age children) and community-based treatment (for under-fives) is implemented twice yearly during the child health days in April and October. However, in schistosomiasis high-risk villages and in lymphatic filariasis (LF) endemic districts, the first round of mass drug administration (MDA) targets the whole population while the second round of treatment is limited to under-fives and school-age children. No study has been done to assess the impact of school-based deworming which has been ongoing since 2004. Program monitoring has been limited to reported treatment coverage, but the reports have never been validated and it is possible that the data are exaggerated.

### Morbidity and burden of STH

Soil-transmitted helminths frequently cause chronic and debilitating diseases, mainly in infants, preschool and school-aged children, adolescent girls and pregnant women [12–13]. Global burden is typically expressed in DALYs estimated as high as 39 million, similar to malaria or tuberculosis[11, 14]. Globally an estimated 807–1221 million people are infected with *A*.*lumbricoides*, 604–795 million with *T*.*trichiura*, 576–740 million with hookworms [12, 15]. Concurrent infections with multiple helminth species are common [16–19] a known cause of malnutrition, intestinal obstruction, biliary colic and pancreatitis[20]. *T*.*trichiura* infections can induce Trichuris dysentery syndrome, whose symptoms include rectal prolapse, anemia, and clubbing of fingers[21]. Hookworm is implicated as the causative factor in more than 50% of cases of iron deficiency anemia in Asia and Africa [22].

Generally, infections with STH have a negative impact on pregnancy and birth outcomes, hamper children’s cognitive and physical development [23–24], resulting in reduced work capacity, and therefore compromise the social and economic development of communities and entire nations [11, 25]. An infection level of *A*.*lumbricoides* and *T*.*trichiura* are highest in children aged between 5 and 14 years [14]with decline in frequency and intensity in adulthood [26]. This age dependency might be due to changes in exposure and/or acquired immunity [10] and morbidity associated with the number of worms harbored[27].

The aim of this study was to evaluate the impact of a national deworming campaign on the prevalence and intensity of soil-transmitted helminthic infections in Uganda.

## Materials and methods

### Study area

**Fig 1:**
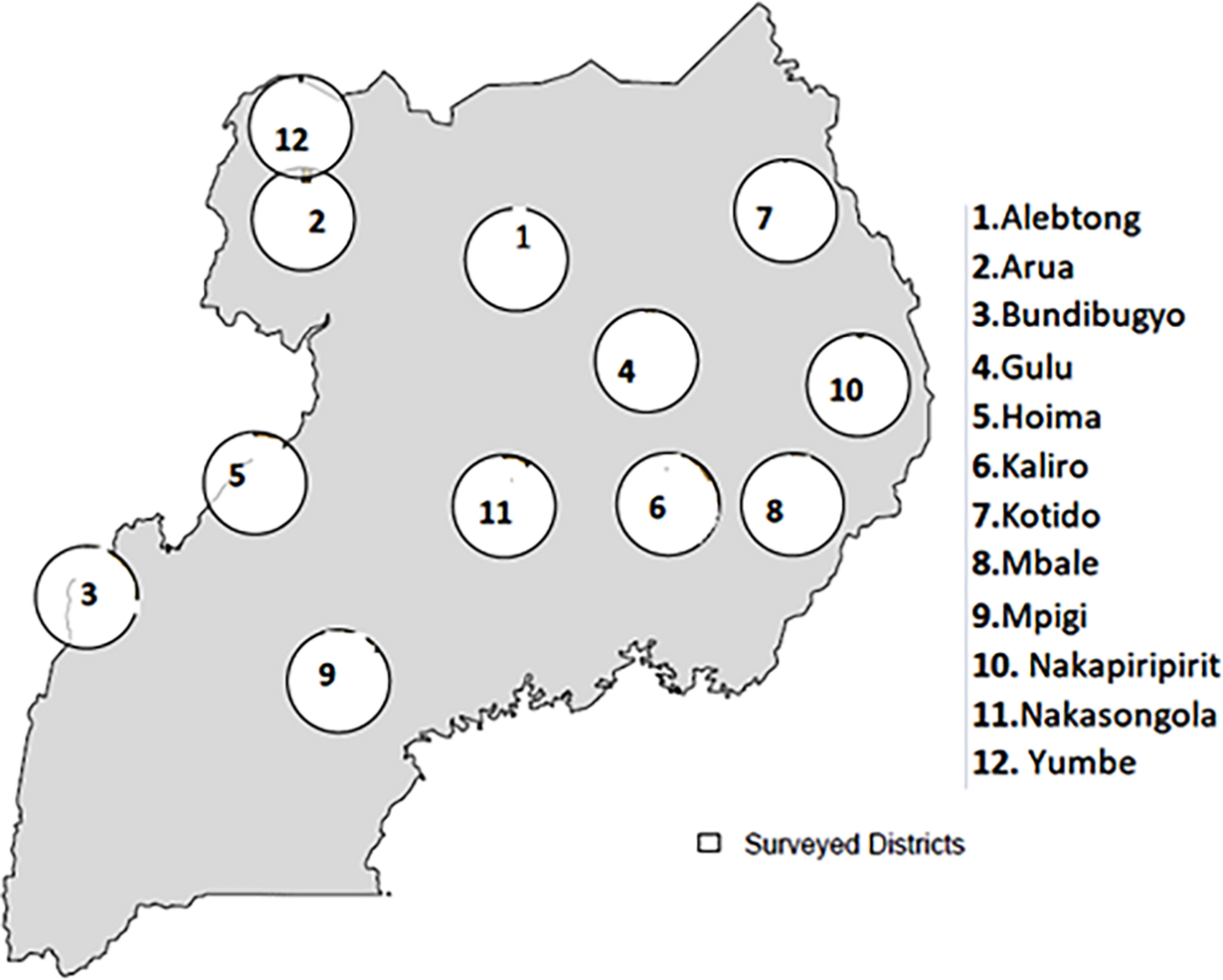
Map showing location of study districts.

The selection criteria for the districts was based on the six regions in which two districts were selected per region with Central region (Mpigi and Nakasongola), Karamoja region(Nakapiripirit and Kotido), West Nile region (Arua and Yumbe), Northern Region (Alebtong and Gulu),Eastern region (Mbale and Kaliro) and Western region (Bundibugyo and Hoima). The Fig 1 below shows the list districts that were selected for the surveys.

### Study design

The study was descriptive and cross-sectional. The major objective of the study was to assess the impact of deworming on STH and guided by the following objectives:

1. to determine the prevalence of all STH species infection among school-age children in identified districts
2. to compare the prevalence of infection before and after interventions
3. To examine the current water and sanitation conditions in schools and relate the parameters to the current prevalence of infection
4. To assess knowledge and practice of the population regarding STH transmission

### Selection of study population and data collection

From each district, five schools were randomly selected. In each of the selected schools, children aged 6–14 years (30 boys and 30 girls) were randomly selected, whereas, in communities, 60 participants aged 5 years and above were selected and conducted during June-July 2016 period.

### Laboratory methods

Duplicate Kato-Katz thick smears [2] were prepared in the field upon receipt of the fecalsamples and were read under a microscope within 60 minutes of slide preparation to determine hookworm ova. To avoid inter-observer errors, the two slides from one specimen were read by different technicians. For quality control, 10% sub-sample of the slides was re-read by an experienced technician. There was no significant difference between the technicians’ readings and those of the sampled slides.

### Measurement of haemoglobin levels

Haemoglobin (Hb) levels were measured using finger prick blood taken from each child for the assessment of Hb as proxy to measuring anaemia levels. The Hb was measured in the field using a portable hamoglobinometer (HemoCue Hb301, LTD Angelholm, Sweden). Determination of anaemia levels followed WHO references defined as: Hb< 115 g/L for children 5–11 years; Hb<120 g/L for children 12–14 years; Hb<120 g/L for non-pregnant women ≥ 15 years; Hb<110 g/L for pregnant women; Hb<130 g/L for men ≥ 15 years [28–29].

### Measuring knowledge, attitude, and practice

A short pretested questionnaire was administered to each participant to obtain their knowledge, attitudes, and practices relating to infection and control of STH. General observations were made on the sanitations of their environments. Observations were made on school and community environment including availability and conditions of latrines and hand washing facilities. Coordinates of each school were taken using a GPS machine (GarminGPSMAP62, Garmin Ltd, Southhampton, UK).

### Treatment and ethical approval

All children found positive for intestinal schistosomiasis (egg-patency) were treated with praziquantel (Distocide, Shin Poong Pharmaceuticals, Seoul Republic of Korea) at 40 mg/kg body weight. Regardless of infection status, a tablet of albendazole (400mg) was given to each child for soil-transmitted helminthiasis. This study was approved by the Ugandan National Council of Science and Technology and formed part of the monitoring and surveillance activities of the Ugandan National Bilharzia & Worm Control Programme. Permission from school authorities was sought and children consented before participation in the study.

### Data analysis

A descriptive analysis of the data on prevalence and intensity of infection from the national registry of the Ministry of Health in 2004 was done and compared with the recent study for the selected districts. Besides the analysis of frequency and proportion distributions, the geographic locations of prevalence and intensity of infection data points for school-age children were mapped, as this was useful to visualize gaps in data. The database was made with MS-Excel 2013 and the analysis with STATAVersion10. All the data were double entered into the computer using excel programme by different clerks.

The impact on the intensity of infection was determined using student’s T-test. Binary logistic regression analysis was utilized to determine WASH factors as associated with STH infections. *Multivariable models* for infection with each type of infection was adjusted for gender, residence in a treatment area, playing barefoot; and reported the presence of latrine and any other purported risk factor under study. None overlapping 95% confidence intervals and p-values <0.05 were considered as significant levels.

## Results

Overall data were collected from a total of 4,275 children including 719(16.8%) from central region, 718(16.8%) from eastern region, 719 (16.8%) from northern region, 689 (18.1%) from Karamoja region, 717(16.7%) from West Nile region and 723(16.9%) from western region. The average age of the children was 12.6 years with a standard deviation, SD 1.8 years and the minimum age was 6 years and upper age limit of 12 years. The percentage of boys (50.2%) and girls (49.9%) was comparable as shown below in Table 1.

**Table 1:** Showing the characteristics of the study participants.

*Variable*	*Categories*	*Total Examined*	*Percentage (%)*
**Regions**	Central	719	16.78
	Eastern	718	16.76
	Northern	719	16.78
	Karamoja	689	16.08
	Westnile	717	16.73
	Western	723	16.87
**Sex**	Male	2,149	50.15
	Female	2,136	49.85
**Age group**	6–9 years	3,844	89.71
	10–14 yrs	441	10.29

### Prevalence of *STH* infections

Table 2 above summarizes the prevalence and intensities of infection by the district. Generally, the pattern of Soil-transmitted helminths (STH) varied markedly with an overall 8.8%, (95% CI; 8.0–9.7) infected with at least any one STH species. The most common soil-transmitted helminths were hookworms (7.7% 95% CI; 6.9–8.5), *Trichuris trichiura* (1.3%, 95% CI; 1.0–1.7) and *Ascaris lumbricoides* (0.5%; 95% CI; 0.3–0.7) respectively. The highest prevalence infection was observed in Bundibugyo district with the prevalence of 24.9% [20.5–29.4], 4.1% [2.1–6.1], 4.1% [2.1–6.1] and 28.2% [23.6–32.8] for *Hookworm*, *Ascaris lumbricoides*, *Trichuris trichiura* and any infections of *STH* respectively. The prevalence of infection for *Hookworm infections* varied from 0.0% in Kotido to -24.9% in Bundibugyo district, while for *Ascaris lumbricoides* ranged from 0.0% in Arua, Gulu, Kaliro, Kotido, Mbale, Nakasongola and Yumbe to 4.1% in Bundibugyo district. A similar trend to *Ascaris lumbricoides* was observed for *Trichuris trichiura infections*.

**Table 2:** Showing the prevalence and intensity of infections for *Hookworm*, *Ascaris lumbricoides*, *Trichuris trichiura and Any STH* infections by district.

District	*Prevalence of STH*				*Hook worm Intensity of Infection*			*Ascaris lumbricoides intensity of Infection*		*Trichuiris trichiura intensity of Infection*		Previous MDA coverage (%)
	*Hookworms (%) [95% CI]*	*Ascaris lumbricoides (%) [95% CI]*	*Trichuiris trichiura (%) [95% CI]*	*Any STH (%) [95% CI]*	*Light* *(1-1999epg)*	*Moderate (2*,*000–3*,*999epg)*	*Heavy (*≥4,000 epg)	*Light* *(1–4*,*999 epg)*	*Moderate* *(5*,*000–49*,*999epg)*	*Light* *(1–999 epg)*	*Moderate* *(1*,*000–9*,*999epg)*	
Alebtong	2.2[0.7–3.8]	0.3[-0.3–0.8]	0.6[-0.2–1.3]	3.1[1.3–4.9]	8 (100%)	0 (0%)	0 (0%)	1(100%)	0(0%)	2(100%)	0 (0%)	59.9
Arua	2.2[0.7–3.8]	0	0.3[-0.3–0.8]	2.5[0.9–4.1]	8(100%)	0 (0%)	0 (0%)	0 (0%)	0(0%)	1(100%)	0 (0%)	41.7
Bundibugyo	24.9[20.5–29.4]	4.1[2.1–6.1]	4.1[2.1–6.1]	28.2[23.6–32.8]	91(100%)	0 (0%)	0 (0%)	14(93.3%)	1(6.67%)	16(100%)	0 (0%)	94.0
Gulu	1.1[0–2.2]	0	0	1.1[0.0–2.2]	4(100%)	0 (0%)	0 (0%)	0(0%)	0(0%)	0(0%)	0 (0%)	100.6
Hoima	9.8[6.7–12.9]	0.6[-0.2–1.3]	2.5[0.9–4.1]	12.0[8.6–15.4]	37(100%)	0 (0%)	0 (0%)	2(100%)	0(0%)	9(100%)	0 (0%)	50.5
Kaliro	21.4[17.2–25.7]	0	0	21.4[17.2–25.7]	77(96.2%)	0 (0%)	3(3.80%)	0 (0%)	0(0%)	0(0%)	0 (0%)	44.2
Kotido	0	0	0	0	0 (0%)	0 (0%)	0 (0%)	0 (0%)	0(0%)	0(0%)	0 (0%)	74.4
Mbale	6.7[4.1–9.3]	0	0.3[-0.3–0.8]	6.7[4.1–9.3]	26(100%)	0 (0%)	0 (0%)	0 (0%)	0(0%)	1(100%)	0 (0%)	66.0
Mpigi	11.7[8.4–15.1]	0.8[-0.1–1.8]	6.7[4.1–9.3]	16.8[12.9–20.6]	40(100%)	2 (4.7%)	1(2.3%)	2(66.67%)	1(33.30%)	23(95.8%)	1(4.2%)	92.7
Nakapiripirit	3.0 [1.2–4.8]	0.3[-0.3–0.9]	0.6]-0.2–1.4]	3.6[1.6–5.6]	6(60.0%)	2 (20.0%)	2 (20.0%)	0 (0%)	1(100%)	2((100%)	0 (0%)	93.1
Nakasongola	8.6[5.7–11.5]	0	0.8[-0.1–1.8]	9.4[6.4–12.4]	6(60.0%)	0 (0%)	0 (0%)	0 (0%)	0(0%)	3(100%)	0 (0%)	50.1
Yumbe	0.3[-0.3–0.8]	0	0	0.3[-0.3–0.8]	31(100%)	0 (0%)	0 (0%)	0 (0%)	0(0%)	0(0%)	0 (0%)	80.9
**Total**	**7.7[6.9–8.5]**	**0.5[0.3–0.7]**	**1.3[1.0–1.7]**	**8.8[8.0–9.7]**	**329[97.1%]**	**4[1.2%]**	**6[1.8%]**	**19[86.36%]**	**3[13.64]**	**57[98.30%]**	**1 (1.70%)**	** **

### Intensity of helminthic infections

Generally, the majority of infections observed across all the districts surveyed had a light intensity of infections with the greatest number of light infection category observed at 57 (98.3%), 329(97.1%) and 19(86.4%) for *Trichuris trichiura infections*, *Hookworm infections*, and *Ascaris lumbricoides* respectively. The prevalence of light intensity of infections for *Hookworm infections* ranged from 0.0% to 100% with majority (97.1%) of infections in this category however; there was a marked reduction in the prevalence of intensities of infections from moderate (1.2%) to heavy (1.8%) intensities of infections observed across all surveyed districts.

The prevalence of light intensities of infections for *Ascaris lumbricoides* varied from 0.0% to 100% with an overall 86.4% while the prevalence of moderate intensities of infections ranged from 0.0% to 100% with an overall 13.6%. There were no individuals observed with heavy intensities of infection. The prevalence of light intensities of infections for *Trichuris trichiura infections* ranged from 0.0% to 100% with an overall 98.3% while Mpigi district recorded 4.2% prevalence for moderate intensities of infections. There were no individuals observed with heavy intensities of infection.

### Comparison of prevalence of *STH* infection at baseline in 2004 and 2016

**Fig 2:**
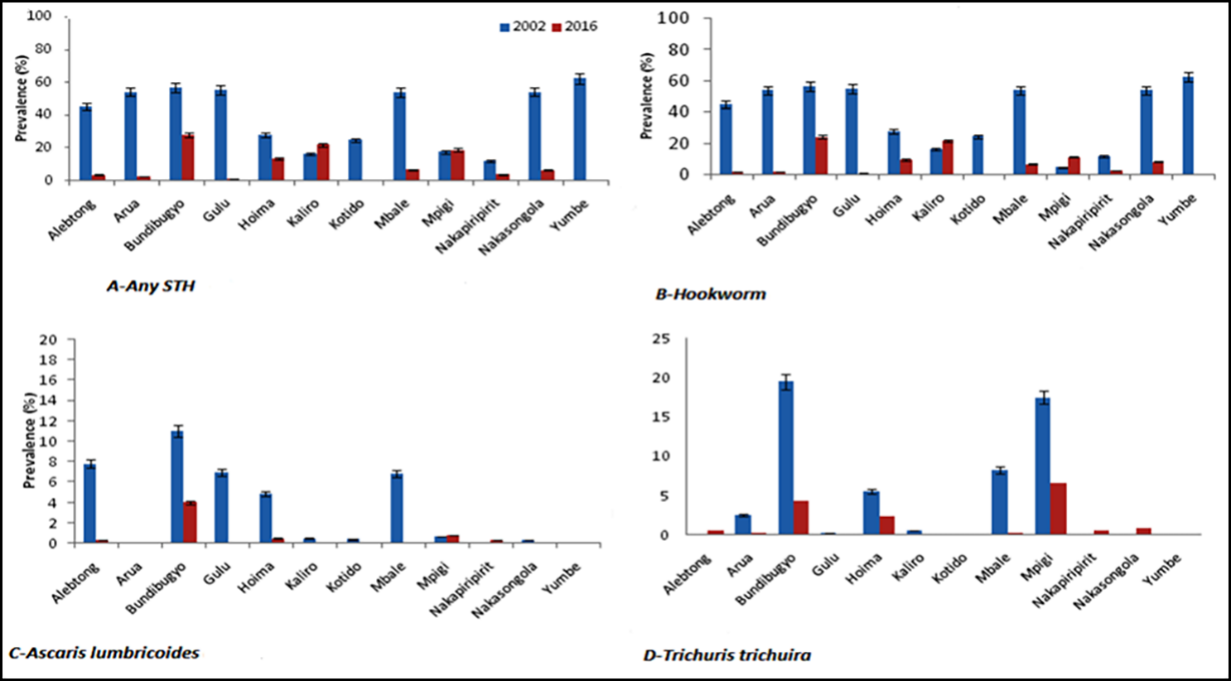
Showing comparisons of the prevalence of infections between baseline 2002 and follow-up in 2016.

Overall, the study results show that the prevalence of STH was generally high at baseline (2002) compared to the findings of this survey in 2016. For example; in Yumbe district that had the highest burden of STH among the districts surveyed in 2002 versus 2016 showed a tremendous reduction from 62.5% to 0.3% (99.5% reduction in prevalence). A similar trend was observed in other districts like; Bundibugyo (56.8% vs. 27.7%), Gulu (55.3% vs. 1.1%), Nakasongola (54.3% vs. 6.6%), Arua (54.3% vs. 2.5%), Mbale (54.1% vs. 6.9%), Alebtong (45.4% vs. 3.6%), Hoima (27.9% vs. 13.2%), Kotido (24.7% vs. 0.3%) and Nakapiripirit (12.2% vs.3.9%). However, there was an increase in STH prevalence observed for Kaliro (16.4% vs. 21.9%) as shown in Fig 2A.

#### Comparison of *Hookworm* infection

Overall, the results show that the prevalence of Hookworm infections was generally high at baseline in 2002 compared to over a decade of Deworming campaign in Uganda that achieved a significant reduction. However, there was a significant increase in Hookworm prevalence in Mpigi (4.9% vs. 11.6%) and Kaliro (16.4% vs. 21.9%) as shown in *Fig 2B*

#### Comparison of *Ascaris lumbricoides* infection

The overall prevalence of *Ascaris lumbricoides* infections was generally high at baseline in 2002 and a significant reduction in prevalence observed in the majority of the districts surveyed in 2016. However, a reverse trend of prevalence (0.7% vs. 0.8%) and (0% vs. 0.3%) was observed for Mpigi and Kaliro districts as shown in *Fig 2C*.

#### Comparison of *Trichuris trichiura* infection

The overall prevalence of *Trichuris trichiura* infections was generally high at baseline in 2002 and a significant reduction in prevalence observed in the majority of the districts surveyed in 2016. However, there was a slight increase in prevalence (0% vs. 0.6%) for Alebtong, (0% vs. 0.6%) for Nakapiripirit and (0% vs. 0.8%) for Nakasongola districts as shown in *Fig 2D*.

### Prevalence of anemia

Overall 22.2% were anaemic with 0.4% having severe anaemia. Gulu district had the highest percentage of individuals with severe anaemia at (56.0% any anaemia vs. 1.7% severe anaemia, 95% CI; 113.2–116), followed by Kaliro district with 2.4% for any anaemia vs. 1.1% severe anaemia, 95% CI; 120.9–132). Other districts with severe cases were Yumbe (37.3% any anaemia vs. 0.3% severe anaemia, 95% CI; 120.3–137.2), Nakasongola (15.6% any anaemia vs. 0.3% severe anaemia, 95% CI; 126–129), Mpigi (6.7% moderate anaemia vs. 0.3% severe anaemia, 95% CI; 131.8–134), Hoima (26.1% any anaemia vs. 0.3% severe anaemia, 95% CI; 123.4–126), Bundibugyo (33.7% any anaemia Vs 0.3% severe anaemia, 95% CI; 118.9–131) and Alebtong (0.6% any anaemia Vs 0.3% severe anaemia, 95% CI; 168.4–172) as shown in Table 3 below. The limitation here is lack of baseline anaemia data for these districts for comparison.

**Table 3:** Prevalence of anaemia by district.

District	No. Examined	No. with anaemia	% Anaemia	% Severe Anaemia	Mean Hb	95% CI
Alebtong	353	2	0.6	0.3	170.3	168.4–172
Arua	359	160	44.6	0.0	119.7	118.3–121
Bundibugyo	365	123	33.7	0.3	124.9	118.9–131
Gulu	357	200	56.0	1.7	114.7	113.2–116
Hoima	360	94	26.1	0.3	124.8	123.4–126
Kaliro	359	89	24.8	1.1	126.5	120.9–132
Kotido	359	0	0.0	0.0	175.9	167.9–184
Mbale	360	66	18.3	0.6	128.8	127.2–130
Mpigi	358	24	6.7	0.3	132.9	131.8–134
Nakapiripirit	329	3	0.9	0.0	167.6	165.6–170
Nakasongola	359	56	15.6	0.3	127.3	126–129
Yumbe	357	133	37.3	0.3	121.7	120.3–123
**Overall**	**4275**	**950**	**22.2**	**0.4**	**136.0**	**134.8–137.2**

### Treatment coverage over the years for *STH*

Mass drug administration (MDA) is the mainstay of morbidity control for schistosomiasis and soil-transmitted helminths (STHs)[5]; hookworm, roundworm, and whipworm are the three main species of STHs that infect humans. MDA is the delivery of free single-treatment preventive chemotherapies at regular intervals to endemic populations. STHs are treated with albendazole (ALB) or mebendazole (MBZ). Repeated annual or biannual treatments are necessary mainly due to susceptibility to reinfection after treatment [30–31]. The central challenges identified by the WHO to increase MDA coverage are sustaining financial support for MDA, improving monitoring and evaluation, expanding local administrative capacity, and increasing access to preventive chemotherapies during MDA[32].

**Fig 3:**
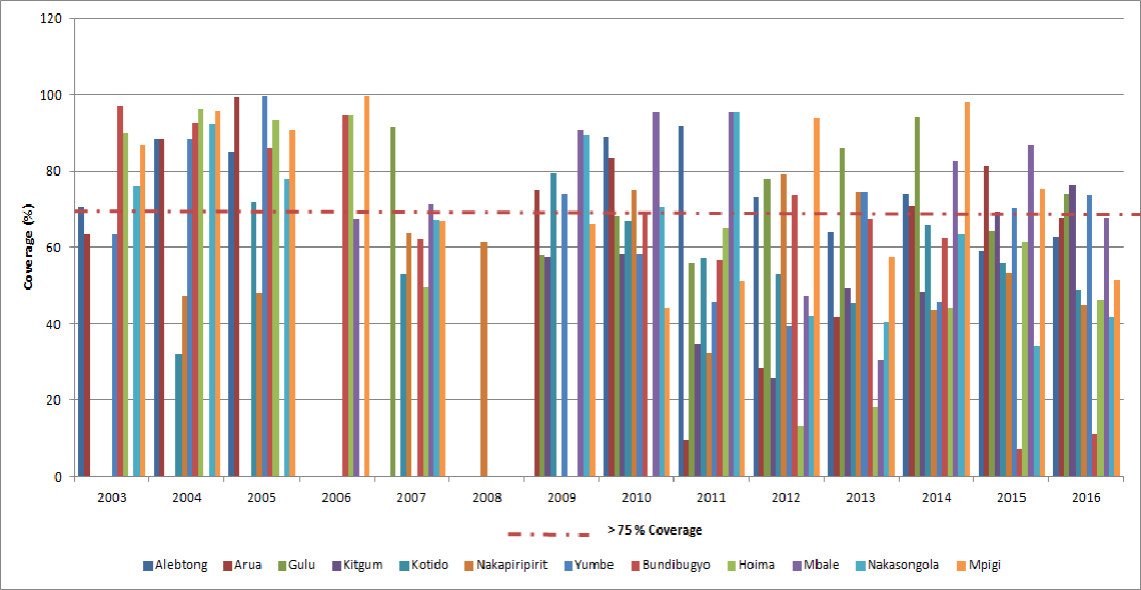
Treatment coverage for the surveyed districts over the years for STH.

Implemented by districts using school teachers and volunteers known as Village Health Teams (VHTs) and Healthcare workers during Child Days plus (CDP) with the aim to control morbidity. The delivery strategy is through mass annual anthelmintic treatment targeted at school-aged children and high-risk groups in the endemic areas using Albendazole (ALB) /Mebendazole (MBZ) to treat STH infection [33–34]. Generally, there has been a tremendous improvement in the STH worm burden over the years. However, there is still a challenge to STH elimination as some districts continue to register lower MDA coverage than recommended >75% by WHO as indicated in Fig 3 below.

### Knowledge, attitude, and practice on the *STH* transmission and prevention

All the children interviewed knew what STH were (62.8%, 95% CI: 61.3–64.2), Table 4. The most common knowledge of information were; home (39.0%, 95% CI: 37.1–40.8), media (radio& newspaper) (11.0%, 95% CI: 9.8–12.2), school (65.7%, 95% CI: 63.9–67.5) and friends (11.5%, 95% CI: 10.3–12.7). Majority of the study participants interviewed were aware of how one gets infected with STH through; eating contaminated food (77.5%, 95% CI: 76.0–79.1), walking barefoot (59.6%, 95% CI: 57.8–61.5), drinking contaminated water (52.9%, 95% CI: 51.0–54.8), playing in dirty places (21.8%, 95% CI: 20.2–23.3) and dirty hands (2.3%, 95% CI: 1.7–2.9).

**Table 4:** Showing the effects of knowledge, attitude, and practice on the *STH* transmission and prevention in 2016.

Variables	Categories	Yes (%) 95% CI	Mean	Std.
Do you know how worm infections are transmitted	Yes	62.8 [61.3–64.2]	0.63	0.01
How did learn about worm infections	From Home	39.0 [37.1–40.8]	0.39	0.01
	Over the Radio/TV/News paper	11.0 [09.8–12.2]	0.11	0.01
	From school	65.7 [63.9–67.5]	0.66	0.01
	From friends	11.5 [10.3–12.7]	0.12	0.01
How does one get infected with worms	Eating contaminated food	77.5 [76.0–79.1]	0.78	0.01
	Walking bare foot	59.6 [57.8–61.5]	0.6	0.01
	Drinking contaminated Water	52.9 [51.0–54.8]	0.53	0.01
	Playing in dirty places	21.8 [20.2–23.3]	0.22	0.01
	Dirty hands	02.3 [01.7–02.9]	0.02	0
How do you prevent worm infections	Washing hands with Soap	69.5 [67.8–71.2]	0.7	0.01
	Covering food	67.0 [65.2–68.8]	0.67	0.01
	Wearing shoes	52.8 [51.0–54.7]	0.53	0.01
	Taking medicines	25.6 [23.9–27.2]	0.26	0.01
Sanitation	Do wash Your hands	91.3 [90.5–92.2]	0.91	0
	How many times a day	70.0 [68.3–71.8]	1.71	0.01
When do you wash your hands?	After using a latrine/toilet	66.5 [65.1–67.9]	0.66	0.01
	Before eating	91.4 [90.5–92.2]	0.91	0
	After eating	78.1 [76.9–79.3]	0.78	0.01
	Before preparing and handling food	14.5 [13.4–15.5]	0.14	0.01
	Finger Nails cut	66.7 [65.3–68.1]	0.67	0.01
How often do you wear shoes?	Occasionally	42.8 [41.3–44.3]	1.81	0.01
Do you wash fruits before eating them?	Always	38.0 [36.6–39.5]	1.71	0.01
Why do you wash hands and fruits	Prevention of diseases	58.1 [0.709–0.736]	0.72	0.01
In the Past 6 months, have you Albendazole/mebendazol?	Yes	72.3 [70.9–73.6]	2.09	0.01
Why were you not treated	Absent	30.6 [27.9–33.3]	2.43	0.03
	Feared side effect	00.9 [00.3–1.4]	0.8	0.02
	Drug not given	65.3 [62.5–68]	1.72	0.03
	Did not feel sick/ill	03.3 [01.8–04.7]	2.69	0.02

## Discussion

Generally, there was a significant reduction in the worm burden throughout the country, although, this could be attributed to regular deworming and improved sanitation over the years. A national deworming program was initiated in 2003 by the Ugandan Ministry of Health (MOH) with support from the Schistosomiasis Control Initiative (SCI) and later scaled-up to the national level in 2005 [6]. A uniform strategy involving school-based and community directed implementation has been implemented in Uganda twice yearly in April and October. However, in Schistosomiasis high-risk villages and in lymphatic filariasis (LF) endemic districts, the first round of mass drug administration (MDA) targets the whole population while the second round of treatment is limited to under-fives and school-age children. Integrating disease control programs can result in improved cost-effectiveness and resource efficiency where endemic populations overlap and more so where MDA and disease-specific requirements are similar. In most communities, the environmental Health Division of MoH has been running a sanitation program and this has contributed to improved sanitation and promotion of safe water supply provision. This is believed to have contributed to reduction in STH infections.

Disease diagnosis is very challenging in a rural context where resources may not be adequate but very important for monitoring program progress in the context of integrated control as programs move towards elimination strategy of 2020. Integrated NTD activities can result in improved cost-effectiveness and resource efficiency, where endemic populations overlap and particularly where MDA and disease-specific assessment requirements are somewhat similar. This study has shown that deworming campaigns in Uganda has achieved significant reduction in disease prevalence for STH over the years for example; Significant reductions were observed in prevalence of Hookworm infections in districts that implement MDA for LF alone for example; Bundibugyo (56.8% vs. 27.7%), Mbale (54.1% vs. 6.9%), Kotido (24.7% vs. 0.3%) and Nakapiripirit (12.2% Vs.3.9%). However, there was an increase in STH prevalence observed for Kaliro (16.4% vs. 21.9%) compared to districts that don’t have LF. It is interesting to note that children who attended school had a lower risk of STH infection, probably because they were more likely to be dewormed than their counterparts that did not attend school and secondly children in schools are congregated resulting into higher treatment impact in school-based deworming. This was observed in similar studies [35].

For districts that are endemic for Lymphatic Filariasis and Onchocerciasis, mass treatment using IVM has been on-going. The use of Ivermectin in the treatment of Onchocerciasis has contributed significantly in the reduction of the burden of soil-transmitted helminths for all districts surveyed in this study except for Mpigi which is neither Lymphatic Filariasis (LF) nor Onchocerciasis program supported district. A number studies [35–36] have shown similar observations where deworming significantly reduces worm burden.

The same study further points out that 17.7% had any type of anaemia, 4.8% had moderate and only 0.6% had severe anaemia and studies have by [37] proved that Antihelminthic treatments are very effective means of improving hemoglobin(Hb) levels although may not be specific to iron deficient anaemia[38].

Our study findings show that awareness and knowledge about soil transmitted helminths has increased unlike in studies by [39] where they observed low awareness and knowledge information among participants for STH.

Majority of the children knew what soil transmitted helminths were (62.8%, 95% CI: 61.3–64.2) and most common knowledge of information were from; home (39%, 95% CI: 37.1–40.8), media (radio& newspaper)(11%, 95% CI: 9.8–12.2), school(65.7%, 95% CI: 63.9–67.5) and friends(11.5%, 95% CI: 10.3–12.7). Majority were aware of how one gets infected with soil transmitted helminths through; eating contaminated food (77.5%, 95% CI: 76.0–79.1), walking barefoot (59.6%, 95% CI: 57.8–61.5), drinking contaminated water (52.9%, 95% CI: 51.0–54.8), playing in dirty places (21.8%, 95% CI: 20.2–23.3) and dirty hands (2.3%, 95% CI: 1.7–2.9).

### Challenge to elimination of *STH* morbidity in Uganda

The target for STH in Uganda is control whereby we aim at eliminating the associated morbidity to levels of no public health importance (WHO 2012 Roadmap for NTDs Implementation towards 2020). Treatment and limited health education alone cannot sustain STH morbidity elimination. The high reproductive capacity of soil transmitted helminthes means that in the absence of additional interventions, transmission cannot be interrupted even if the infection intensity is greatly reduced [40] and this can lead to morbidity recrudescence. There is need for stable provision and use of adequate sanitation facilities to end open defecation, improved personal and food hygiene behavior and access to clean water [41].The Uganda sanitation and water supply have made substantial progress since 1990s especially in urban centers (http://en.wikipedia.0rg/wiki/water). The government with support from multilateral and bilateral agencies, NGOs and the private sector has been vigorously supporting programmes aimed at improving safe water supply for now over 3 decades. In early 1990s, only 10% of urban dwellers were supplied with safe water but the figure had risen to 81% by 2006. However, most households and schools rural areas in Uganda still have inadequate sanitation and safe water supply. When the whole population estimated at 37.5 million people is considered, access to safe water was still just 62% [42] and has improved a little since then.

Despite efforts punt into improving sanitation, it has been accorded low priority with little resources and inadequate collaboration and coordination among stakeholders [43]. According to Ministry of Health records, the national average toilet coverage is still at around 68% but it varies; 80% in urban areas, 60% in rural areas and about 40% in slums [43]. However, latrine coverage in some communities can be as low as 10% especially in fish landing villages [44]. In some rural areas like Karamoja and the Lake Victoria Islands, superstitions against using toilets result in extensive open air defecation [43]. There is almost complete absence of hand washing facilities [43–44] and all these factors promote STH transmission.

### Conclusions

The situation as it is today shows reduced levels of STH morbidity. This status cannot be guaranteed unless sanitation issues are well addressed. Without significantly improving WASH, it will be difficult to interrupt STH transmission in most parts of the country. It is also necessary to prioritize operational research agenda that includes NTD monitoring within the local government health sector. Nationally, there is need to monitor STH programme every 5 years to guide the MoH on feasible interventions. Routine evaluations should not be limited to impact on infection rates but should also aim at evaluating impact of the programme on nutrition and educational achievements. This would leverage proper resource allocation and utilization. Combating the burden of STH requires a holistic approach and it should be a responsibility of all stakeholders including the affected communities.
